# CAR-NK cell therapy: promise and challenges in solid tumors

**DOI:** 10.3389/fimmu.2025.1574742

**Published:** 2025-04-07

**Authors:** Sahar Balkhi, Gaia Zuccolotto, Anna Di Spirito, Antonio Rosato, Lorenzo Mortara

**Affiliations:** ^1^ Immunology and General Pathology Laboratory, Department of Biotechnology and Life Sciences, University of Insubria, Varese, Italy; ^2^ Immunology and Molecular Oncology Unit, Veneto Institute of Oncology IOV - IRCCS, Padova, Italy; ^3^ Department of Surgery, Oncology and Gastroenterology, University of Padova, Padova, Italy

**Keywords:** cellular immunotherapy, CAR-T therapies, natural killer (NK) cells, CD19-CAR-NK, CAR-NK therapies

## Abstract

Over the past few years, cellular immunotherapy has emerged as a promising treatment for certain hematologic cancers, with various CAR-T therapies now widely used in clinical settings. However, challenges related to the production of autologous cell products and the management of CAR-T cell toxicity highlight the need for new cell therapy options that are universal, safe, and effective. Natural killer (NK) cells, which are part of the innate immune system, offer unique advantages, including the potential for off-the-shelf therapy. A recent first-in-human trial of CD19-CAR-NK infusion in patients with relapsed/refractory lymphoid malignancies demonstrated safety and promising clinical activity. Building on these positive clinical outcomes, current research focuses on enhancing CAR-NK cell potency by increasing their *in vivo* persistence and addressing functional exhaustion. There is also growing interest in applying the successes seen in hematologic malignancies to solid tumors. This review discusses current trends and emerging concepts in the engineering of next-generation CAR- NK therapies. It will cover the process of constructing CAR-NK cells, potential targets for their manufacturing, and their role in various solid tumors. Additionally, it will examine the mechanisms of action and the research status of CAR-NK therapies in the treatment of solid tumors, along with their advantages, limitations, and future challenges. The insights provided may guide future investigations aimed at optimizing CAR-NK therapy for a broader range of malignancies.

## 1 Introduction

Cancer is a highly heterogeneous and complex disease, representing a leading cause of mortality globally, with increasing incidence and mortality rates annually. It encompasses a vast array of malignancies originating from virtually every type of cell in the body, making it a significant challenge for medical research and clinical treatment. Traditionally, the mainstays of cancer treatment have been surgery, radiation therapy, and chemotherapy. While these methods have proven effective in many cases, particularly for localized tumors, they come with notable limitations. The effectiveness of these traditional approaches diminishes significantly in the context of metastatic or recurrent cancers, where the likelihood of achieving long-term survival remains low. Additionally, these treatments often lack specificity, resulting in substantial collateral damage to healthy tissues and leading to severe side effects (1–3).

Over the past decade, our growing understanding of the molecular mechanisms driving cancer has led to the development of targeted therapies. These therapies are designed to specifically target molecular pathways or genetic alterations that are unique to cancer cells, thereby minimizing damage to normal cells. One of the most groundbreaking advancements in this realm is immunotherapy, which aims to harness and enhance the body’s immune system to recognize and destroy cancer cells more effectively. Immunotherapy has revolutionized the landscape of cancer treatment, offering a novel approach that goes beyond the limitations of conventional therapies (4–6).

One of the most promising immunotherapeutic strategies is the development of Chimeric Antigen Receptor (CAR) therapies. Among these, Chimeric Antigen Receptor (CAR) T-cell therapy has emerged as a promising approach. CAR T-cell therapy involves genetically modifying α/β T cells to express chimeric antigen receptors (CARs) that recognize cancer-specific antigens. This therapy can be administered in autologous or allogeneic settings. In the autologous approach, T cells are extracted from the patient, engineered to express CARs, expanded, and reinfused, reducing immune rejection risks but potentially limited by the patient’s health. The allogeneic method uses T cells from healthy donors, modified to mitigate graft-versus-host disease (GvHD) and immune rejection, allowing for “off-the-shelf” CAR T-cell therapies with faster availability and consistent quality (7).

The generation of CAR T cells takes place in specific and accredited laboratories that adhere to strict Good Manufacturing Practice (GMP) standards. These facilities ensure quality control, regulatory compliance, and standardized production processes to enhance the safety and efficacy of CAR T-cell therapies (8).

CAR-T cell therapy has demonstrated remarkable success in the treatment of hematological malignancies, particularly B- cell leukemias and lymphomas. For instance, CAR-T cells targeting the CD19 antigen on B cells have achieved complete remission rates of up to 85% in patients with acute lymphoblastic leukemia (ALL) and up to 100% in patients with refractory or relapsed B-cell acute lymphoblastic leukemia (r/r B- ALL) (9–11). The success of CAR-T cell therapy has generated considerable excitement and optimism in the field of cancer immunotherapy, leading to its approval for clinical use in several hematological cancers. However, its application in solid tumors has proven to be far more challenging. CAR T therapy shows promise for hematologic malignancies but faces challenges in solid tumors due to the tumor microenvironment (TME). Irregular tumor vasculature hinders CAR T-cell extravasation, as endothelial cells often lack adhesion molecules necessary for transmigration. The dense extracellular matrix (ECM), enriched with collagen and fibronectin, further restricts T-cell movement, while cancer-associated fibroblasts (CAFs) exacerbate ECM stiffness. Hypoxia in the TME stabilizes HIFs in tumor-associated macrophages (TAMs), promoting pro-tumor factors that impede CAR T-cell infiltration. Overcoming these barriers is key to improving CAR T-cell efficacy in solid tumors (12, 13).

Additionally, solid tumors often exhibit heterogeneity in antigen expression, allowing them to escape immune surveillance by losing or downregulating the targeted antigen, a phenomenon known as immunoediting. This leads to antigen loss or antigenic variability, reducing the effectiveness of CAR-T cell therapy (14).

Moreover, CAR-T cell therapy is associated with a range of potential side effects, some of which can be severe and life-threatening. On-target, off-tumor toxicity occurs when CAR-T cells recognize and attack normal cells expressing the target antigen, leading to unintended damage to healthy tissues. Another significant adverse event is cytokine release syndrome (CRS), a systemic inflammatory response triggered by the rapid activation and proliferation of CAR-T cells. CRS can result in high fever, hypotension, and multi-organ dysfunction, necessitating prompt medical intervention. Neurotoxicity, which can manifest as confusion, seizures, or cerebral edema, is another serious complication associated with CAR-T cell therapy. These adverse events highlight the need for careful patient monitoring and management during and after treatment (15, 16).

Given the limitations and challenges associated with CAR-T cell therapy, researchers have been actively exploring alternative immune effector cells that can be engineered with CARs to enhance antitumor responses while minimizing side effects. One such promising alternative is the use of Natural Killer (NK) cells. NK cells are a type of innate immune cell that play a critical role in the body’s first line of defense against infections and malignancies. Unlike T cells, NK cells can recognize and kill cancer cells without prior sensitization or antigen specificity, making them an attractive platform for CAR engineering (17).

CAR-NK cell therapy offers several potential advantages over CAR-T cell therapy. Firstly, NK cells have a lower risk of causing severe side effects such as cytokine release syndrome (CRS) and neurotoxicity. This is because NK cells predominantly produce a different set of cytokines compared to T cells, which are less likely to trigger the systemic inflammatory responses associated with CRS. Additionally, NK cells do not cause graft-versus-host disease (GVHD), making them safer for allogeneic use. This means that CAR-NK cells can be derived from healthy donors or established cell lines, enabling the development of “off-the-shelf” products that are readily available and can be administered to patients without the need for individualized manufacturing (18–20).

Furthermore, NK cells possess an inherent ability to target and kill cancer cells through a variety of mechanisms, including direct cytotoxicity, antibody-dependent cellular cytotoxicity (ADCC), and the release of cytotoxic granules. This multi-faceted approach allows CAR-NK cells to target cancer cells more effectively, even in cases where antigen expression is heterogeneous or downregulated. The potential for combination therapy with other treatments, such as immune checkpoint inhibitors or conventional therapies, further enhances the therapeutic promise of CAR-NK cells, providing a more comprehensive and robust approach to cancer treatment (21, 22).

Current research is focused on optimizing CAR-NK cell therapy for clinical use, with several preclinical and early-phase clinical trials demonstrating promising results. For example, CAR-NK cells targeting the B-cell maturation antigen (BCMA) have shown efficacy in preclinical models of multiple myeloma, and CAR-NK cells targeting HER2 have demonstrated antitumor activity in models of HER2-positive solid tumors. While these studies highlight the potential of CAR-NK cell therapy, further research is needed to evaluate their ability to penetrate solid tumors effectively, as the TME presents significant barriers to treatment, similar to those faced by CAR-T cell therapies (23, 24). While cancer remains a formidable challenge with high morbidity and mortality rates, recent advancements in immunotherapy, particularly CAR-based therapies, have opened new avenues for treatment. CAR- NK cell therapy emerges as a compelling alternative for CAR-T therapy, offering a safer profile, innate antitumor capabilities, and the potential for off-the-shelf use. As research progresses, CAR-NK cell therapy and other innate immune cell-based approaches hold the promise of revolutionizing cancer treatment, offering a new generation of therapies that are safer, more effective, and adaptable to a broader range of cancers. This review will focus on the role of CAR-NK cells in the treatment of solid tumors and explore the recent advances that are shaping the future of this promising therapeutic modality.

## 2 NK Cells

### 2.1 Human NK cells: classification and development

NK cells are a part of the innate lymphoid cell family and are primarily found in lymph nodes, bone marrow, blood, lungs, spleen, and liver. In human peripheral blood, bone marrow, and tissues, NK cells are identified by the absence of surface TCR and CD3 molecules and by the expression of neural cell adhesion molecule (NCAM; also known as CD56). Additionally, NK cells can be identified by the expression of the natural cytotoxicity receptor 1 (NCR1), also known as NKp46 (or CD335), a receptor specifically associated with NK cell function and activation (25).

NK cells derive from multipotent CD34+ hematopoietic progenitors in the bone marrow. Unlike T cells, their maturation occurs in the bone marrow and lymphoid organs without requiring the thymus (26). The turnover of human NK cells in blood is approximately 2 weeks, with an estimated doubling time of 13.5 days. Even when differentiation from progenitor cells is impaired, NK cells persist in the peripheral blood, suggesting homeostatic maintenance. NK cells have a proliferative potential lower than that of T cells, limited by telomere shortening, but overexpression of telomerase reverse transcriptase (TERT) has shown to significantly extend their lifespan (27, 28).

NK cells are categorized into CD56bright and CD56dim subsets based on CD56 expression levels. The majority (>90%) of NK cells in peripheral blood are CD56dim, known for their high cytotoxic potential and significant role in tumor immunotherapy. CD56bright NK cells are either precursors to CD56dim cells or act as effector cells with lower cytotoxicity. However, they produce cytokines, growth factors, and chemokines to regulate immune responses. CD56dim NK cells, in contrast, have strong natural cytotoxicity and antibody-dependent cell-mediated cytotoxicity, primarily targeting tumor cells, virus- infected cells, and parasites (29–31).

### 2.2 Memory and functional adaptations in NK cells

Traditionally, it was believed that NK cell responses were not enhanced by repeated exposure to the same target. However, emerging evidence indicates that NK cells can acquire ‘memory-like’ functional features. This adaptation is characterized by heightened functional activity, rapid release of IFN-γ, and the ability to generate specific recall responses. In humans, CMV infection has been shown to result in an increase in the proportion of NK cells expressing the activating receptor NKG2C, which recognizes CMV peptides (32). Memory NK cells in humans are identified by markers such as CD57, NKG2C, CD62L, EOMES, and T-bet, which distinguish them from naive NK cells. The expression of KLRG1 is linked to terminal differentiation, whereas EOMES and T-bet regulate their development. In CMV infection, KLRG1^−Ly49H^+ naive NK cells preferentially generate memory NK cells, while KLRG1^+ cells form short-lived effectors. Both intrinsic factors, such as RAG expression during NK cell ontogeny, and extrinsic factors like microbiota-derived signals and T cell interactions influence NK cell maturation and memory formation. Furthermore, transcription factors such as EOMES and T-bet play crucial roles in the development and differentiation of memory NK cells, regulating their functional maturation and persistence. These factors promote the ability of memory NK cells to respond quickly to re-infection (33).

Proteomic analyses of memory NK cells from humans have shown upregulation of proteins involved in fatty acid metabolism, DNA repair, and mitochondrial function, contributing to their survival and cytotoxic potential. Additionally, memory NK cells demonstrate enhanced metabolic fitness, relying on both glycolysis and oxidative phosphorylation (OxPhos) to meet energy demands during rapid expansion and effector function. Epigenetic changes regulate these metabolic pathways, establishing the memory characteristics of NK cells. Memory NK cells also exhibit altered receptor profiles, including the expression of inhibitory receptors like NKG2A, which helps regulate their response to self-MHC class I molecules, and CXCR6, which directs them to key tissues such as the liver, spleen, and lymph nodes. Their enhanced ability to rapidly produce cytokines such as interferon-γ (IFN-γ) and cytotoxic molecules like perforin and granzyme B allows them to effectively eliminate tumor cells and virus-infected cells (34, 35).

### 2.3 NK cells’ mechanisms for targeting and killing tumor cells

NK cell activation is managed by a suite of activating, co-stimulatory, and inhibitory receptors. These receptors determine whether an adjacent cell is targeted for killing and regulate cytokine secretion. A fundamental function of NK cells is the elimination of cells with diminished or absent expression of major histocompatibility complex (MHC) class I molecules. During development, the interaction between killer cell immunoglobulin-like receptors (KIRs) and self-MHC molecules provides essential signals for NK cell maturation and contributes to their functional competency, a process known as ‘licensing’. Moreover, Mature NK cells are suppressed by intact self-MHC ligation but are activated if MHC is altered or downregulated, a common feature of tumor cells. Additionally, tumor cells often exhibit the upregulation of stress-induced antigens, such as MICA and MICB, which are recognized by the activating receptor NKG2D, further enhancing NK cell-mediated cytotoxicity (36, 37).

NK cells eliminate tumors through several pathways:

1. Granule Exocytosis: Releasing perforin and granzyme-containing granules to induce apoptosis in tumor cells. Granzymes enter the target cells and trigger apoptosis, while perforin forms pores in the cell membrane.
2. Death Receptor-Mediated Apoptosis: Secreting tumor necrosis factor (TNF) family members like FasL and TRAIL, which bind to receptors on target cells and trigger apoptosis.
3. Cytokine and Chemokine Secretion: Inducing tumor cell death by restricting angiogenesis and boosting adaptive immunity through the release of anti-cancer effector molecules like interferon-g (IFN-g). Cytokines such as IL-2, IL-12, IL-15, and IL-18 enhance NK cells’ anti-tumor effects.
4. Antibody-Dependent Cell-Mediated Cytotoxicity (ADCC): NK cells express Fc receptors (CD16) that bind to the Fc region of antibodies. CD16 engagement by immunoglobulin-opsonized cells induces a signaling cascade, resulting in the killing of the antibody-coated cell (38).

### 2.4 Immune escape mechanisms of tumor cells

Despite the crucial role of NK cells in immune surveillance, tumor cells can evade this surveillance through various strategies. Tumor cells may reduce the expression of ligands recognized by NK cell receptors via mechanisms such as metalloproteinase-mediated cleavage or excretion in exosomes. Chronic stimulation by activating ligands can induce resistance in NK cells by reducing the expression of adaptor molecules like DAP10 and DAP12. Additionally, tumor cells can overexpress ligands for inhibitory NK cell receptors, such as HLA-E, which binds to the inhibitory CD94-NKG2A receptor complex, thereby suppressing NK cell activity. While downregulation of HLA-I occurs in some tumors, most tumor cells express sufficient HLA-I to inhibit NK cells, and chronic inflammation within the tumor microenvironment can further induce HLA-I expression via IFN-γ, contributing to NK cell resistance (39, 40)

Soluble factors in the tumor microenvironment, such as TGF-β, can suppress NK cell activation by blocking the mTOR signaling pathway triggered by IL-15, reducing NK cell proliferation and cytotoxicity. Other factors like prostaglandin E2, l-kynurenine, adenosine, and lactic acid can also dampen NK cell activation (41). Additionally, tumor cells promote an immunosuppressive response by stimulating regulatory T cells (Tregs) and recruiting myeloid-derived suppressor cells (MDSCs), which impair NK cell function through inhibitory cytokines and metabolic disruption. Cancer-associated fibroblasts (CAFs) exacerbate this by recruiting Tregs and M2 macrophages and producing extracellular matrix components that hinder NK cell infiltration (42).

Understanding the intricate biology of NK cells, including their development, memory capabilities, activation, and mechanisms for targeting tumor cells, as well as the strategies employed by tumors to evade NK cell-mediated destruction, is critical for advancing cancer immunotherapies.

## 3 Construction of CAR-NK cells

Constructing Chimeric Antigen Receptor Natural Killer (CAR-NK) cells is a sophisticated process involving several intricate steps, each critical to ensuring that these engineered cells effectively target and destroy cancer cells. The construction and optimization of CAR-NK cells combine molecular engineering with cellular immunology, resulting in a powerful therapeutic approach with the potential to significantly impact cancer treatment (43).

At the core of CAR-NK cell construction is the design of the Chimeric Antigen Receptor (CAR). This receptor is a synthetic molecule designed to endow NK cells with the ability to specifically recognize and attack cancer cells. The CAR structure is typically composed of several essential domains: the ectodomain, the hinge region, the transmembrane domain, and the intracellular signaling domains (44).

The ectodomain is the extracellular component of the CAR and is primarily responsible for recognizing and binding to specific antigens present on the surface of target cancer cells. This part of the CAR often includes a Single-Chain Fragment Variant (scFv), which is a fusion protein derived from the variable regions of both the heavy and light chains of an antibody. The scFv is crucial because it determines the specificity of the CAR for its target antigen. To construct an effective scFv, researchers must select an appropriate antibody and optimize its binding affinity for the target antigen. This involves engineering the scFv to ensure that it can effectively bind to the cancer cell antigen while avoiding normal tissues. Additionally, the orientation of the variable domains (VH-VL or VL-VH) can affect antigen recognition and binding affinity. Most research favors the VH-VL orientation due to its generally favorable performance, although both configurations can be effective depending on the context (45, 46).

Connecting the scFv to the rest of the CAR structure is the hinge region. This region, sometimes referred to as the spacer, links the scFv to the transmembrane domain and provides flexibility and stability to the CAR. The hinge region must be long enough to allow the scFv to engage with its target antigen effectively, especially when the antigen is located at varying distances from the cell membrane. Commonly used hinge regions include those derived from CD28, immunoglobulin, or CD8α domains. Each of these hinge regions has unique properties that can influence the CAR’s performance. For instance, CD28-derived hinge regions might enhance the activation signal, but they could also lead to increased risk of side effects such as cytokine release syndrome. In contrast, immunoglobulin-based hinge regions provide greater flexibility and stability, which can be beneficial for maintaining proper CAR function (45, 47–49).

**Figure 1:**
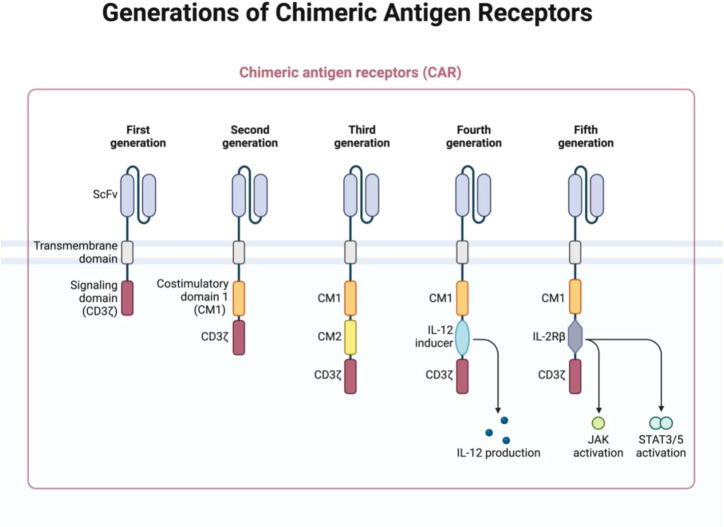
The structure of chimeric antigen receptors (CARs) has advanced through several generations, each defined by unique features. The first generation included only ITAM motifs of the CD3ζ chain in the intracellular domain, which limited signaling capacity. Second-generation CARs introduced one co-stimulatory molecule (CM), enhancing the cell function, while the third generation added an additional CM for greater efficacy. The fourth generation built upon the second by including 1–3 ITAMs and the inducible or constitutive expression of cytokines like IL-12. The fifth generation, or “next generation,” is also based on second-generation CARs but incorporates cytokine receptor intracellular domains, such as the IL-2Rβ chain with STAT3/5 binding sites, to further enhance signaling. (Figure created with BioRender).

The transmembrane domain anchors the CAR to the NK cell membrane and plays a critical role in linking the extracellular antigen-binding domain to the intracellular signaling components. This domain is essential for ensuring that the CAR is properly integrated into the cell membrane and that it effectively transmits signals from the scFv binding event to the intracellular signaling domains. Common choices for the transmembrane domain include those derived from CD3ζ, CD8, and CD28. Each of these domains influences the CAR’s stability and signaling capacity. For instance, CD8- derived transmembrane domains might promote CAR dimerization and interaction with endogenous T cell receptors, while CD28-derived domains can enhance the overall activation signal (50). The intracellular signaling domains are pivotal for translating antigen-binding events into functional responses within NK cells. These domains typically include an activation signal derived from CD3ζ, which initiates the cytotoxic response. Second-generation CARs improve upon first-generation designs by incorporating costimulatory domains, such as CD28 or 4-1BB (CD137), which enhance NK cell activation, proliferation, and persistence. These costimulatory signals synergize with the activation signal to boost CAR-NK cell efficacy. However, while third-generation CARs integrate multiple costimulatory signals, evidence suggests that excessive signaling can be counterproductive. Overactivation resulting from the combined use of CD28 and 4-1BB in third-generation CARs has been shown to accelerate apoptosis, increase exhaustion, and impair proliferative capacity in T cells. This phenomenon, likely due to activation-induced cell death (AICD) and excessive costimulatory signaling, underscores the complexity of designing optimal CAR structures (51). Importantly, studies indicate that the effectiveness of costimulatory domains depends on factors such as the scFv affinity, antigen expression levels, and target tumor type. Therefore, while third-generation CARs aim to enhance NK cell functionality through multiple costimulatory signals, their design must carefully balance stimulatory strength to avoid detrimental effects like exhaustion. Future CAR constructs should focus on tailoring intracellular domains to the specific needs of the target antigen and tumor environment to optimize both signaling intensity and cellular responses (52–55). The vector backbone used to introduce the CAR gene into NK cells is another critical component. This vector must include a promoter, which regulates the expression of the CAR gene. Various promoters, such as EFS (Elongation Factor-1 alpha), PGK (Phosphoglycerate Kinase), and CMV (Cytomegalovirus), can be

used to drive CAR expression. The choice of promoter affects the level of CAR expression and, consequently, the therapeutic efficacy of the CAR-NK cells. For instance, EFS and PGK promoters are known for their ability to maintain high levels of gene expression over time, which is important for ensuring sustained CAR expression in NK cells. The vector also includes polyadenylation signals that help stabilize the CAR mRNA, ensuring efficient translation and protein production (**Figure 1**) (56–59).

An essential consideration in CAR-NK cell construction is the efficiency of CAR expression on the surface of NK cells. This process involves selecting appropriate signal peptides (SPs), which are short peptide sequences that guide the CAR to the endoplasmic reticulum and Golgi apparatus for proper processing and membrane integration. The signal peptide is crucial for ensuring that the CAR is correctly assembled and expressed on the NK cell surface. Commonly used signal peptides include those derived from CD8α and immunoglobulin chains. These peptides are designed to facilitate the proper folding and transport of the CAR protein, although the optimal signal peptide for CAR-NK cells is still an area of ongoing research (60–62).

The insertion of the CAR gene into the NK cell genome is a critical factor in determining the success of CAR-NK cell therapy. Traditional retroviral vectors integrate randomly into the genome, posing risks such as insertional mutagenesis and the potential generation of replication-competent retroviruses (RCRs). While lentiviral vectors (LVVs) have been considered safer, concerns regarding insertional oncogenesis persist. However, comprehensive studies, including large-scale clinical trials monitored by the National Gene Vector Biorepository (NGVB), have demonstrated no evidence of RCR in patients treated with retroviral gene therapies​. To enhance safety, advances in LVV design have focused on engineering packaging cell lines and incorporating self-inactivating long terminal repeats (SIN-LTRs), which significantly reduce the likelihood of RCR formation. The NGVB’s extensive screening of gene therapy products using sensitive biological assays and qPCR has confirmed the absence of RCRs in a wide range of trials​. Additionally, regulatory measures, such as stringent vector production standards and post-treatment monitoring, have further reinforced the safety profile of retroviral and lentiviral gene therapy​. These innovations ensure that LVVs remain a reliable tool for CAR-NK cell therapies, addressing previous safety concerns while maintaining efficient gene transfer capabilities. The rigorous testing and regulatory frameworks now in place provide strong evidence supporting the continued use of LVVs in clinical applications with minimal risk of RCR-related complications (63, 64).

Recent advancements in CAR-NK cell technology have introduced innovative strategies to enhance the specificity, safety, and efficacy of these therapies. One such strategy involves the use of logic gate systems, which enable CAR-NK cells to respond only to specific combinations of antigens. This approach helps reduce off-target effects and improves the precision of the therapy. For example, “AND” logic gates require the simultaneous recognition of two antigens for CAR activation, enhancing specificity and reducing the risk of damaging normal tissues (65–67). Similarly, “AND- NOT” logic gates incorporate both activating and inhibitory CARs to prevent damage to healthy tissues by distinguishing between tumor and non-tumor antigens (68, 69).

Adaptor-dependent CARs represent another innovative approach, where external ligands or small molecules are used to activate the CAR. This method provides additional control over CAR-NK cell activity and allows for targeting multiple antigens. Pharmacologic switches, which can turn CAR activity on or off using small molecules, offer a mechanism to manage adverse effects and improve safety. These switches include on-switch systems like Tet-ON, which uses doxycycline to induce CAR

expression, and off-switch systems like the SWIFF CAR, which uses drugs or genetic modifications to control CAR activity or induce cell death in case of severe adverse effects (70–74).

Enhancing CAR-NK cell efficacy can also be achieved by arming the cells with additional therapeutic molecules. For example, Bispecific T-cell Engagers (BiTEs) are engineered molecules containing two scFv fragments linked by a flexible peptide, enabling simultaneous binding to tumor antigens and CD3 on T cells. This promotes targeted immune responses and enhances CAR cell activity. Cytokines like IL-12 or IL-15 can be engineered into CAR-NK cells to enhance their persistence and anti-tumor effects. ECM-degrading enzymes like heparanase can improve tumor infiltration, and exosomes can be used to deliver therapeutic RNA or other molecules to enhance tumor responses.

### 3.1 Cell sources for CAR-NK generation

**Figure 2:**
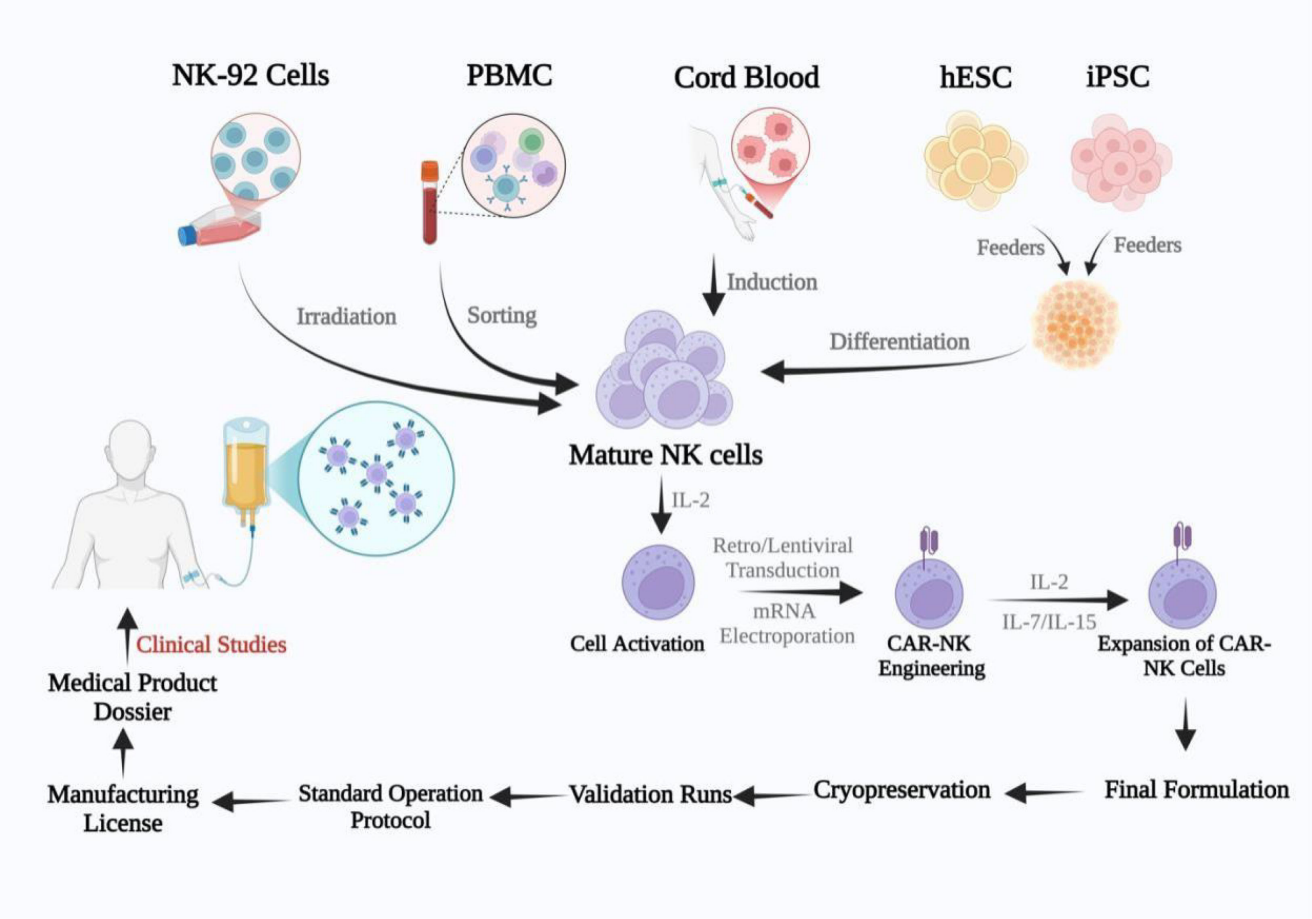
Sources and workflow of chimeric antigen receptor-natural killer (CAR-NK) cell therapy. NK cells can be sourced from NK-92 cell lines, peripheral blood mononuclear cells (PBMC), umbilical cord blood (UCB), and embryonic stem cells (ESC)/induced pluripotent stem cells (iPSC). These cells are activated using IL-2 and transduced with a CAR construct, followed by ex-vivo expansion and quality control. After cryopreservation and validation, CAR-NK cells are prepared for clinical infusion to target and eliminate tumor cells (Figure created with BioRender).

Generating CAR-NK cells for immunotherapy requires a large number of these cells, which can be challenging due to their limited lifespan and proliferative capacity. Various sources are used to isolate and produce enough NK cells for CAR-NK applications (**Figure 2**). The following explores the benefits and drawbacks of these different sources.

#### 3.1.1 Peripheral blood NK cells

NK cells are relatively easy to isolate from either the patient (autologous PB-NK) or healthy donors (allogeneic PB-NK), with most preclinical CAR-NK studies using PB-NK, particularly from healthy donors. Autologous NK cells are often less effective due to functional silencing by self-MHC antigens or compromised function from the patient’s disease or prior treatments, such as intense cycles of chemotherapy and radiotherapy, which can weaken and reduce the quality of the lymphocytes, including NK cells. These prior treatments can impair the number and functionality of NK cells, limiting the success of autologous therapies. Therefore, allogeneic PB-NK cells are usually preferred for therapy. However, obtaining sufficient NK cells is challenging due to their low numbers in peripheral blood, necessitating extensive expansion. Although PB-NK cells are mature and do not require long differentiation processes, they face issues with low transduction efficiency and potential exhaustion from prolonged culture, despite advancements in stimulatory molecule combinations (75–77).

#### 3.1.2 Cord blood-derived NK cells

PB-NK cells rely on donor availability, which can be problematic in emergency situations. In contrast, cord blood provides a ready alternative with its high NK cell yield. For example, a small fraction of a cord blood unit can generate over a billion NK cells within two weeks. Additionally, the haplotype of cord blood can be determined at collection, allowing the creation of a cell bank with HLA-mismatched options. While CB-NK cells may have lower expression of certain cytotoxicity receptors compared to

other NK cells, they have still shown promise in clinical trials, with recent studies demonstrating their effectiveness and long-term persistence *in vivo* (78–81).

#### 3.1.3 Stem cell-derived NK cells

Using human embryonic stem cells (hESC) or induced pluripotent stem cells (iPSC) to derive NK cells offers a standardized, off-the-shelf product, reducing variability between donors. Although production from these stem cells takes 3-5 weeks, which is longer than other methods, it provides a more consistent product. iPSC-derived NK cells have shown functionality against tumors and have been used to generate CAR-NK cells. However, there are concerns about potential malignant transformation and immunogenicity, as well as the dependency on continuous cytokine administration for cell proliferation (50, 82, 83).

#### 3.1.4 NK cell lines: NK-92

NK cell lines like NK-92 simplify the process of obtaining large numbers of NK cells, as they are easy to maintain and expand. Derived from lymphoma, NK-92 cells show strong anti-tumor activity and can be genetically modified. However, they are tumor-derived and aneuploid, requiring irradiation before infusion to prevent *in vivo* proliferation, limiting their persistence. Clinical trials suggest NK-92 cells are safe and effective, but their inability to proliferate *in vivo* may affect long-term efficacy compared to primary NK cells (84, 85). However, in clinical settings, especially in Europe, their use faces limitations. K562 cells, used to expand NK cells, have tumorigenic properties, raising safety concerns. Residual K562 cells may remain in the final product, requiring extensive purification, which complicates the manufacturing process and increases costs. European regulatory bodies, like the EMA, impose strict standards for cell-based therapies, making it difficult to use tumor-derived feeder cells like K562 in clinical protocols (86, 87).

Alternative NK cell expansion methods, such as artificial antigen-presenting cells (aAPCs) or feeder-free systems, are being explored to enhance safety and align with regulatory standards, supporting clinical application (88). Ongoing research into these methods is vital for integrating NK cell therapies into clinical practice.

#### 3.1.5 NK cell expansion

To achieve the required number of NK cells for clinical use, various expansion methods are employed. These include using cytokine mixtures, K562 cell-based systems with membrane-bound cytokines, and advanced automated expansion technologies. Although expansion methods have improved, achieving clinically relevant numbers can still take several weeks, and optimizing transduction efficiency while maintaining cell viability remains a challenge. Methods like co-culturing with K562 feeder cells have shown promise, with high transduction efficiency and relatively quick expansion times (88, 89).

### 3.2 Transfection or transduction vehicle for CAR expression

With advances in gene modification technologies, several methods have been developed for generating CAR-NK cells, primarily focusing on viral transduction or transfection. Two predominant approaches are viral transduction, using lentiviruses or retroviruses, and transfection methods like naked plasmid DNA, transposase DNA-mediated integration, or mRNA via electroporation.

Lentiviruses have been a staple in gene therapy for years due to their ability to transduce both cycling and non-cycling cells efficiently. They integrate into the host genome, providing permanent transgene expression, and have low immunogenicity. Out of 14 reports on primary CAR-NK cells and 44 on CAR-NK cell lines, lentiviruses have been successfully used. Second-generation lentiviral vectors have been common, though third-generation vectors are now preferred due to their increased safety profile. Despite their advantages, lentivirus transduction efficiency in NK cells often remains below 10%, with potential improvements through changes in virus pseudotypes or cytokine stimulation (90, 91).

Retroviruses have also been used extensively for gene therapy, requiring actively dividing NK cells for effective integration. There are 19 studies on CAR-NK cell lines and 16 on primary NK cells using retroviruses. Recent Phase I trials with retrovirus-transduced CAR-NK cells showed promising results, with a high response rate and stable CAR DNA copy numbers in patients for up to a year. Alpha retroviruses with the RD114 envelope have demonstrated superior transduction efficiency compared to gamma retroviruses and lentiviruses (20, 92, 93).

Electroporation of mRNA Electroporation of mRNA has become a key technique for engineering CAR-modified NK cells for adoptive immunotherapy, enabling efficient CAR expression and targeted tumor recognition. This method temporarily permeabilizes cell membranes using an electric field to introduce CAR-encoding mRNA, offering a non-viral, safer alternative to viral vectors by avoiding genome integration and reducing insertional mutagenesis risk (94). The transient nature of CAR expression minimizes long-term adverse effects, offering a controlled therapeutic window. However, electroporation can impact cell viability and the efficiency of mRNA uptake, with optimal conditions needed to balance transfection efficiency and cell survival (95).

#### 3.2.1 Sleeping beauty transposon

The Sleeping Beauty (SB) transposon system is an emerging non-viral approach for engineering CAR NK cells, providing an alternative to viral vectors. It integrates transgenes using a “cut-and-paste” mechanism facilitated by SB transposase and inverted terminal repeats. However, integration occurs semi-randomly rather than in strictly targeted sites, necessitating further investigation (96).

Concerns regarding transposon-based gene transfer arise from findings by Bishop et al., who reported malignant CAR T-cell tumors in two patients treated with piggyBac-modified CD19 CAR T-cells. One patient succumbed to the secondary tumor, while the other achieved remission following additional treatment, highlighting the potential oncogenic risks of transposon-based integration (96).

Despite these concerns, recent research by Bexte et al. demonstrated the successful engineering of CAR NK cells using the SB transposon system with minicircle DNA vectors. These modified NK cells exhibited stable CAR expression, preferential integration into genomic safe harbors compared to lentiviral vectors, and enhanced cytotoxicity against target cells. Additionally, the SB system avoids viral components, reducing immune responses and offering improved transgene control, which may enhance safety (97).

Further research and clinical trials are essential to fully assess the safety and efficacy of the SB system and establish standardized protocols for CAR NK cell therapies.

CRISPR/Cas9 is a cutting-edge technique for genetic modification, offering precise gene editing capabilities. Initially used to disrupt genes like CD38 to prevent NK cell fratricide, CRISPR/Cas9 can also introduce new genes using a homologous donor DNA template. This method has shown high

knock-in efficiencies and the potential for creating NK cells with enhanced anti-tumor properties and longer persistence. Though efficiency in fresh NK cells remains low, ongoing improvements could make CRISPR/Cas9 a powerful tool for generating CAR-NK cells with durable and potent anti-tumor effects (98–100).

## 4 Preclinical and clinical applications of CAR-NK therapy

CAR-NK are emerging as a promising alternative to CAR-T cell therapy, particularly in cancer treatment. Unlike CAR-T cells, which are associated with significant risks such as cytokine release syndrome (CRS) and graft-versus-host disease (GvHD), CAR-NK cells demonstrate strong anti-tumor activity while minimizing these adverse effects. The unique properties of NK cells, such as their innate ability to target tumor cells and their reduced likelihood of causing severe immune-related toxicities, make them an attractive platform for engineering CAR-based therapies. Research on CAR- NK cells, both preclinical and clinical, is still in its infancy, but the potential of this therapy has driven a surge in research and clinical trials aimed at validating its efficacy and safety in treating various cancers, including both hematological malignancies and solid tumors (14, 101, 102).

Preclinical models have showcased the ability of CAR-NK cells to effectively target both hematologic malignancies and solid tumors. Hematologic tumor antigens such as CD19, CD22, BCMA, and CD33 are frequently used as targets in CAR-NK cell therapies. For solid tumors, antigens like NKG2D ligands, PD-L1, ROBO1, and 5T4 have shown promise. These tumor-associated antigens provide a focused approach to tumor targeting, potentially reducing the risk of off-target effects that can lead to severe toxicities in patients (103).

As summarized in **Supplementary Table 1**, clinical trials involving CAR-NK cells are progressing steadily. Over 20 trials are currently ongoing, recruiting, or under evaluation, targeting a diverse range of cancer-specific antigens such as Claudin6, NKG2D ligands, DLL3, and ROBO1. These studies aim to assess the safety, tolerability, and preliminary efficacy of CAR-NK therapies across various cancers, including ovarian, colorectal, and hepatocellular carcinoma, as well as glioblastoma and prostate cancer. The majority of these trials remain in the early stages, with most classified as Phase 1 or Early Phase 1. Some trials, such as NCT05410717 and NCT04847466, have progressed to Phase 2, investigating outcomes like safety, objective response rates (ORR), disease control rates (DCR), and overall survival (OS). A notable trend is the expanding focus on solid tumors, with trials employing novel targets like Claudin6 (NCT05410717), mesothelin (ChiCTR2100048100), and DLL3 (NCT05507593). Additionally, innovative combinations such as PD-L1 CAR-NK cells with immune checkpoint inhibitors (NCT04847466) and IL15-transduced CAR-NK cells (NCT05703854) are under investigation. While still predominantly in the exploratory phase, the growing number of CAR-NK clinical trials underscores the increasing interest and potential of this therapeutic modality in oncology. One of the significant advantages of CAR-NK cells in preclinical settings is their favorable safety profile. This is because NK cells are naturally allogeneic, meaning they can be derived from unrelated donors without the need for strict human leukocyte antigen (HLA) matching. This characteristic enables the development of ‘off-the-shelf’ CAR-NK therapies, which can be pre-manufactured and stored for use in multiple patients. This eliminates the need for patient-specific cell manufacturing, thereby significantly reducing both production time and costs, as there is no need for individualized cell processing or extensive preparation for each patient (104). Despite the promise shown in preclinical models, several challenges remain. NK cells typically have a short lifespan *in vivo*, lasting only about two weeks. Enhancing their persistence and expansion is crucial for achieving durable clinical responses. Researchers are exploring strategies such as incorporating cytokine support, like interleukin-15 (IL-15), into CAR constructs to promote the sustained survival of NK cells post-infusion. Additionally, advances in gene editing techniques are being used to enhance the efficacy of CAR-NK cells, such as by knocking out inhibitory receptors that limit NK cell function (105, 106). Additionally, one of the most significant clinical studies to date is a phase 1/2 trial conducted by Rezvani et al. in 2020. This trial involved the use of allogeneic umbilical cord blood (UCB)-derived CAR-NK cells targeting CD19 in patients with CD19-positive lymphoid malignancies. Out of 11 patients, eight (73%) responded positively to the treatment, and seven achieved complete remission. Importantly, none of the patients developed CRS, neurotoxicity, or GvHD, highlighting the excellent safety profile of CAR-NK therapy. These findings mark a critical step forward in demonstrating the clinical potential of CAR-NK cells, especially in hematological cancers (20). Another notable clinical study involved the use of FT596, an allogeneic induced pluripotent stem cell (iPSC)-derived CAR-NK product developed by Fate Therapeutics. In a phase 1 trial, FT596 exhibited strong anti-tumor activity in patients with relapsed or refractory B-cell lymphoma (r/r BCL), without any reported adverse effects. The use of iPSC-derived NK cells holds promise for the development of scalable, off-the-shelf therapies that can be readily available for cancer patients (107). Moreover, CAR-NK92 cells, derived from the NK92 cell line, have been evaluated in clinical trials for their potential to treat various cancers. The NK92 cell line offers advantages such as ease of expansion and cytotoxic activity. In one trial, HER2-specific CAR-NK92 cells were administered to patients with recurrent glioblastoma, demonstrating a favorable safety profile with no observed side effects during the 24-week post-injection period (108). These early clinical trials underscore the potential of CAR-NK therapy to provide effective cancer treatment with a superior safety profile compared to CAR-T cells. However, challenges remain, particularly in enhancing the persistence and long-term efficacy of CAR-NK cells. One major limitation observed in trials with NK92 cells is their limited *in vivo* persistence, which has prevented the achievement of durable remissions. Irradiated CAR-NK92 cells, for example, were found to lose detectability within one-week post-infusion, limiting their long-term therapeutic potential (109). To address the challenge of NK cell persistence, researchers are developing CAR constructs that include cytokine support, such as IL-15, to enhance the survival and proliferation of NK cells. In the UCB-derived CAR-NK study mentioned earlier, the inclusion of constitutive IL-15 support promoted sustained *in vivo* persistence, with CAR- NK cells persisting for up to a year in responders (24). Additionally, strategies such as incorporating suicide genes into CAR constructs, which allow for the selective elimination of CAR-NK cells in case of adverse reactions, are being explored to further enhance the safety of CAR-NK therapy (110).

## 5 The role of CAR-NK therapy in different types of solid tumors

### 5.1 Expanding CAR-NK therapy to solid tumors

While most clinical trials to date have focused on hematologic malignancies, there is growing interest in expanding CAR-NK therapy to solid tumors. Solid tumors present unique challenges, such as a hostile tumor microenvironment that can suppress immune cell activity and limit the infiltration of immune cells into the tumor mass. However, CAR-NK cells offer several advantages over CAR-T cells in the context of solid tumors. NK cells are naturally capable of homing to tumor sites and exerting cytotoxic effects without the need for prior antigen sensitization, which is a significant limitation of T cells (111).

**Figure 3:**
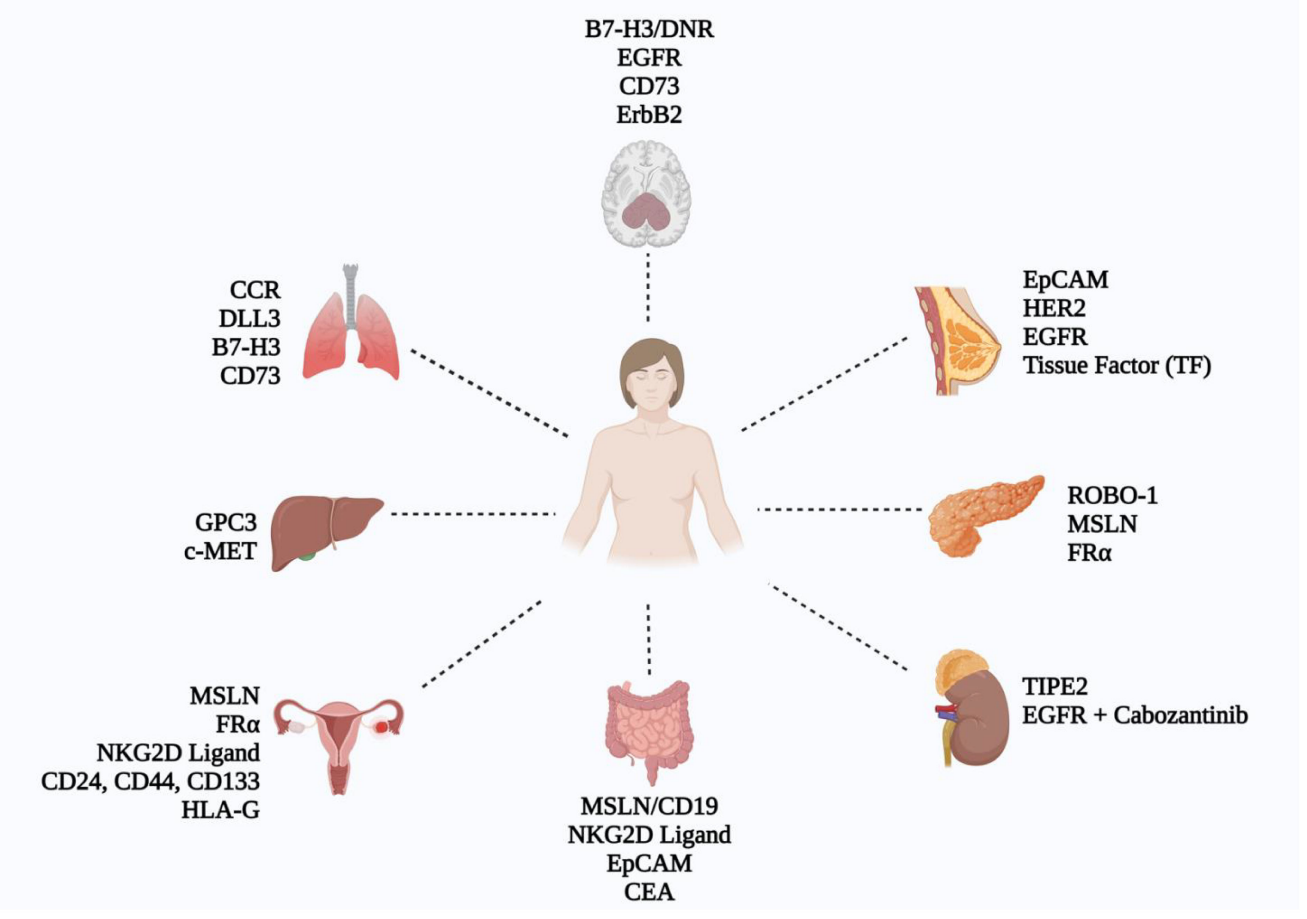
Schematic representation of CAR-NK cell research across various solid tumors, showcasing key targets identified in current CAR-NK studies (Figure created with BioRender).

Several clinical trials are currently investigating the use of CAR-NK cells in solid tumors (**Figure 3**, **Supplementary Table 1**). The following paragraphs will explain recent clinical trials of CAR-NK therapy in various types of solid tumors in more detail.

#### 5.1.1 Glioblastoma

Glioblastoma multiforme (GBM) is the most aggressive brain tumor, with a poor prognosis and an average survival time of only about 15 months despite surgical resection, radiation, and chemotherapy. The tumor microenvironment (TME) in GBM, with its immunosuppressive properties, hinders effective treatment, particularly by inhibiting natural killer (NK) cell activity. However, approximately 89% of GBM tumors naturally contain NK cells, offering a potential avenue for CAR-NK cell therapy (112, 113). Recent studies highlight the promising potential of CAR-NK cells in GBM treatment. Engineered NK cells targeting epidermal growth factor receptor (EGFR) and its variant EGFRvIII have shown extended survival, enhanced cytotoxicity, and increased interferon-gamma secretion in xenograft models (114). Further, genetically engineered NK cells like CD73.mCAR pNK, which target autophagy pathways, improve homing to GBM tumors, enhancing both tumor infiltration and TME modification to make it more receptive to immunotherapy (115). Additionally, NK-92/5.28.z cells targeting HER-2 demonstrated significant anti-GBM activity *in vitro* and in xenograft models (24) (**Supplementary Table 1**). Other targets being explored include IL-13Ra2 and immune checkpoint receptors such as B7-H3 and TGF-β DNR. For example, CAR-NK cells targeting B7-H3 showed significant cytotoxicity against GBM cells (116). In a study with nine GBM patients, NK cell infusion resulted in partial or mixed responses in four patients, underlining the potential but also the need for optimization (113). Innovative approaches such as dual-targeting (e.g., EGFR and EGFRvIII) have shown superior anti-tumor effects by preventing antigen escape, a common issue in CAR-T cell therapies. For instance, DAP12.CD3.CAR-NK-YTS cells targeting EGFRvIII demonstrated improved survival and inhibited tumor growth in xenografts (117, 118). The integration of multiple targeting strategies and TME reprogramming is crucial for enhancing CAR-NK therapy’s efficacy in GBM, and ongoing clinical trials will be essential to evaluate its role in treatment.

#### 5.1.2 Ovarian cancer

Ovarian cancer is an aggressive malignancy with a poor prognosis and a five-year survival rate under 40% (119). Due to its immunogenic features, including significant NK cell infiltration, ovarian cancer has been a key focus for CAR-NK cell therapy (120). Key targets for CAR-NK cells include mesothelin and folate receptor alpha (FRα), both of which are overexpressed in ovarian cancer. Studies have demonstrated that mesothelin-targeted CAR-NK cells effectively eliminate mesothelin-positive ovarian cancer cells (120) (**Supplementary Table 1**). Similarly, FRα-targeted CAR-NK cells showed selective cytotoxicity against FRα-positive cells in xenograft models (121). Additionally, NKG2D-CAR-induced NK cells incorporating a costimulatory domain exhibited significant and robust anti-tumor activity, leading to enhanced tumor clearance and prolonged survival in preclinical models of tumors expressing NKG2D ligands, including ovarian cancer, glioblastoma, and multiple myeloma (50). Cancer stem cell markers such as CD24, CD44, and CD133 are also being explored for CAR-NK targeting. CAR-NK cells targeting mesothelin and FRα have shown enhanced anti-tumor activity both *in vitro* and *in vivo* (116, 122–124). Combining low-dose chemotherapy with HLA-G-targeted CAR-NK cells has also shown promise in increasing cytotoxicity, providing a potential strategy for clinical applications (125). These findings indicate significant potential for CAR-NK cells targeting mesothelin, FRα, and cancer stem cell markers in ovarian cancer therapy. However, clinical trials are needed to validate these promising preclinical results and assess the safety and efficacy of CAR-NK cell therapy in ovarian cancer patients (126).

#### 5.1.3 Breast cancer

CAR-NK cell therapy, have also opened new avenues for treating breast cancer. This therapeutic approach is particularly crucial for aggressive subtypes such as triple-negative breast cancer (TNBC), which often resist conventional treatments. Although numerous studies have demonstrated the potential of CAR-NK cells against various targets, clinical trials specifically focused on CAR-NK therapy in breast cancer are still in their early stages, with ongoing investigations assessing their safety and efficacy (127).

One of the notable targets for CAR-NK therapy in breast cancer is the epithelial cell adhesion molecule (EpCAM). Studies have shown that CAR-NK cells co-expressing CAR and interleukin-15 (IL-15) can sustain proliferation without the need for exogenous cytokines. These engineered NK cells exhibit selective cytotoxicity against EpCAM-expressing breast cancer cells, suggesting their viability for targeted therapy (128).

Another critical target is the human epidermal growth factor receptor 2 (HER-2), which is overexpressed in a significant percentage of breast cancer cases. Clinical studies have utilized HER-2 CAR-NK cells, showing enhanced anti-tumor activity against HER-2-positive breast cancer cells. In particular, NK-92/5.28z cells engineered to target HER-2 have demonstrated substantial cytotoxicity in both *in vitro* and *in vivo* models. These CAR-NK cells maintain high levels of cytotoxic function even in the presence of immunosuppressive elements commonly found in the tumor microenvironment, highlighting their potential utility in solid tumor treatment (129).

In TNBC, where conventional therapies often fail, CAR-NK cells targeting the EGFR have emerged as a promising alternative. Preclinical research indicates that EGFR-CAR-NK cells can effectively lyse TNBC cells and enhance the production of cytokines such as IFN-γ. These findings suggest that CAR- NK therapy could be particularly beneficial for patients with elevated EGFR expression in their tumors (130). Several other antigens have also been explored in the context of CAR-NK therapy for breast cancer. Tissue factor (TF), which is present in a substantial number of TNBC cases, has been targeted using CAR constructs in NK-92MI cells. Modifying these cells to express CD16 has improved their ability to mediate ADCC against TF-expressing breast cancer cells in preclinical models (131).

#### 5.1.4 Lung cancer

Recent advancements in CAR-NK cell therapies have also shown promising results in the treatment of lung cancer, specifically targeting various mechanisms of tumor progression. In a study, Lu developed a chimeric co-stimulatory transition receptor (CCCR) that integrates the PD-1 extracellular domain with NKG2D components, effectively converting negative PD-1 signals into activation signals and reversing immunosuppressive effects. In a lung cancer xenograft model, CCCR-NK92 cells (a third-generation CAR) demonstrated substantial tumor growth inhibition. However, Dr. Zhang et al. noted that this regimen could lead to CRS, necessitating clinician awareness during CAR- NK treatments (132). For non-small cell lung cancer (NSCLC), Liu engineered DLL3-specific NK-92 cells and explored their effectiveness against small cell lung cancer (SCLC). Co-culturing DLL3 SCLC cells with DLL3-CAR NK-92 cells resulted in significant cytotoxicity and cytokine production *in vitro*, while DLL3-CAR NK-92 cells induced tumor regression in a lung metastatic model with an acceptable safety profile (133). In a separate study, Yang demonstrated that NK-92MI cells with an anti-B7-H3 CAR effectively limited the growth of transplanted non-small cell lung cancer in mice, notably extending survival compared to unmodified NK-92MI cells. Increased secretion of perforin/granzyme B and CD107a expression was observed in these modified cells. In another study, Chambers et al. designed a CAR-NK targeting the CD73 adenosine axis, which inhibited adenosinergic metabolism in an NSCLC model, induced tumor stasis, and promoted NK cell infiltration into CD73-expressing tumors (134).

#### 5.1.5 Liver and pancreatic cancer

Pancreatic ductal adenocarcinoma (PDAC) and hepatocellular carcinoma (HCC) are two highly challenging cancers to treat, primarily due to their immunosuppressive TME. In PDAC, researchers have identified roundabout homolog 1 (ROBO-1) as a promising target. Studies in mouse models showed that ROBO-1-targeted CAR-NK cells, combined with brachytherapy, significantly reduced tumor volume. Three ongoing phase I/II trials are assessing ROBO-1-directed CAR-NK therapy in PDAC and other cancers (135–138) (**Supplementary Table 1**). Additional targets include FRα and death receptors 4 and 5 (DR4/5), with studies showing that CAR-NK cells targeting these receptors effectively induce apoptosis in pancreatic cancer cells (139) Furthermore, mesothelin-targeted CAR-NK cells, when co-cultured with the STING agonist cGAMP, showed enhanced anti-cancer effects, both killing cancer cells and boosting immune responses (140). In liver cancer, glypican-3 (GPC3) has emerged as a key target for CAR-NK therapy in HCC. GPC3-specific CAR-NK cells showed strong cytotoxicity *in vitro* and *in vivo* (141), while c-Met-targeted CAR-NK cells demonstrated enhanced cytotoxicity against c-MET-expressing HepG2 cells (142). Ongoing studies are crucial to further explore these promising therapeutic strategies.

#### 5.1.6 Gastric and colon cancer

CAR-NK cell therapies have also shown significant potential in targeting gastric and colon cancers, focusing on specific tumor markers and enhancing NK cell cytotoxicity. In a study conducted by Cao, they engineered MSLN and CD19-targeted CAR NK-92 cells that demonstrated MSLN-CAR NK cells can selectively kill MSLN+ gastric cancer cells while sparing MSLN- cells. These CAR-NK cells effectively eliminate gastric cancer cells in various tumor models and significantly prolong survival in intraperitoneal tumor-bearing mice. Notably, robust antitumor effects and considerable NK cell infiltration were observed in patient-derived xenografts treated with MSLN-CAR NK cells (143). In another study, Xiao et al. constructed an NKG2D RNA CAR (second-generation CAR) using RNA electroporation, which improved NK cell cytotoxicity against several solid tumor cell lines *in vitro*. Three patients with metastatic colorectal cancer received localized infusions of NKG2D RNA-CAR NK cells, resulting in reduced ascitic fluid production and significant tumor cell reduction in samples (144). Moreover, in a separate study, Zhang et al. created an EpCAM-specific second-generation CAR- NK-92 that effectively recognized EpCAM-positive colorectal cancer cells and released key cytokines, showcasing specific cytotoxicity *in vitro*. Shiozawa et al. developed an anti-carcinoembryonic antigen (CEA)-CAR NK-92MI, achieving enhanced cytotoxicity against CEA-positive colon cancer cell lines (145).

#### 5.1.7 Renal cancer

The application of CAR-NK in urological tumors remains limited and is an underexplored area. Tumor necrosis factor-α-inducible protein 8-like 2 (TIPE2) has been identified as a negative regulator of innate and acquired immunity, and its expression correlates with poor prognosis in various tumors, including renal cancers. This may present an opportunity for CAR-NK targeting in renal cancer treatments. Zhang constructed an EGFR-specific third-generation CAR, confirming the specific killing ability of CAR-modified NK-92 cells against renal cell carcinoma (RCC) cell lines. They also investigated the synergistic effects of cabozantinib combined with EGFR-specific CAR-NK-92 cells *in vitro* and *in vivo*, marking a breakthrough in CAR-NK therapy for kidney cancer (146, 147).

## 6 Challenges and potential solutions

### 6.1 Insufficient infiltration

Similar to CAR-T cells, CAR-NK cells encounter challenges in penetrating solid tumors, leading to numerous efforts aimed at enhancing their infiltration. In a study conducted by Temme et al., they found that anti-EGFRvIII CAR-NK cells that overexpressed CXCR4 exhibited increased movement towards U87-MG cells that produce CXCL12, which is a ligand for CXCR4 (118). Likewise, anti- NKG2D CAR-NK cells with elevated CXCR1 expression displayed enhanced movement and infiltration towards hypopharyngeal and ovarian cancer cells in a mouse model (148). Additionally, incorporating oncolytic viruses (OVs) might improve the infiltration of CAR-NK cells, although further investigation is necessary Moreover, local injection methods are being utilized to overcome the significant barriers that restrict infiltration and could enhance the effectiveness of CAR-NK cell therapy (114, 149). In a glioblastoma xenograft mouse model, Yu et al. demonstrated that intracranial injection of EGFR-targeting CAR-NK92 cells effectively inhibited tumor growth and notably increased the survival rate of mice with tumors. Similarly, local injections of HER2-targeting CAR- NK92 cells in glioblastoma mouse models led to complete regression of tumors in most of the mice (24).

### 6.2 Poor CAR- NK *in vivo* persistence

A significant challenge that hampers the effectiveness of CAR-NK cell therapies is their poor persistence within the human body. Unlike T cells, which can develop into long-lived memory cells, NK cells typically have a short lifespan of approximately two weeks post-infusion. This limited persistence poses substantial obstacles for achieving sustained anti-tumor responses and improving patient outcomes (150).

The short-lived nature of NK cells results in a decline in their availability to combat tumors shortly after infusion. Studies have indicated that CAR-NK cells derived from cell lines can proliferate indefinitely *in vitro*; however, their tumorigenic potential necessitates irradiation prior to infusion. This treatment effectively prevents uncontrolled growth but simultaneously limits the cells’ ability to persist and expand *in vivo*. For instance, a clinical trial involving CD33-targeting CAR-NK cells demonstrated that although patients could receive high doses of CAR-NK92 cells without severe side effects, these cells became undetectable merely one week after administration. The transient nature of their presence directly correlates with unfavorable prognoses for patients, as insufficient persistence leads to a lack of long-term remission (109).

To address this critical issue, various strategies have been proposed to enhance the survival and efficacy of CAR-NK cells *in vivo*. One promising approach is the administration of exogenous cytokines, such as interleukin-2 (IL-2) and interleukin-15 (IL-15), which can promote NK cell proliferation and activation. However, the systemic application of these cytokines comes with potential safety risks, including adverse effects associated with high-dose IL-2 therapy, such as vascular leak syndrome. Thus, while cytokines can be effective in extending the lifespan and functionality of NK cells, careful consideration must be given to the associated risks (151).

Another potential solution involves employing lymphodepleting conditioning regimens before the adoptive transfer of CAR-NK cells. By reducing the recipient’s immune cell population, these regimens can help mitigate the risk of rejection and create a more favorable environment for the infused CAR-NK cells. Combinations of agents such as cyclophosphamide and fludarabine are often used as pretreatments to enhance the effectiveness of allogeneic CAR-NK cell therapies. Moreover, multiple infusions of CAR-NK cells may be explored to sustain therapeutic benefits, although the inherent variability of the immune system necessitates rigorous monitoring to prevent immune rejection (152, 153).

The introduction of genetic modifications to enhance NK cell function also shows promise. For example, researchers have developed CAR-NK cells that express IL-15 or incorporate suicide switches, allowing for better control over their persistence and activity. Such modifications not only enhance the cells’ anti-tumor efficacy but also facilitate prolonged survival *in vivo*. Additionally, using CRISPR-Cas9 technology to knock down negative immune checkpoint genes has been shown to improve the durability and anti-tumor activity of CAR-NK cells (154, 155).

The concept of inducing memory-like characteristics in NK cells further contributes to overcoming persistence challenges. Certain cytokines, particularly IL-12, IL-15, and IL-18, can stimulate NK cells

to acquire memory-like properties, enabling them to mount enhanced responses upon re-encountering the same tumor antigens. This memory-like activation could extend the longevity of NK cells in the body and improve their effectiveness in targeting tumors over time (87, 156).

The TME presents an additional challenge to CAR-NK cell persistence. Tumors often produce immunosuppressive factors, including transforming growth factor-beta (TGF-β) and interleukin-10 (IL-10), which can significantly hinder NK cell function. Furthermore, regulatory cells, tumor- associated macrophages, and myeloid-derived suppressor cells infiltrate the TME, contributing to an environment that actively suppresses immune responses. Addressing these barriers through combination therapies that include checkpoint inhibitors and targeting immunosuppressive factors in the TME may enhance the persistence and functionality of CAR-NK cells (157). While strategies such as exogenous cytokine support, genetic modifications, lymphodepleting regimens, and the induction of memory-like properties present potential solutions, addressing the complexities of the TME remains critical.

### 6.3 Tumor microenvironment inhibition

The tumor microenvironment (TME) poses significant challenges for CAR-NK cell therapies due to various immunosuppressive factors, including cancer-associated fibroblasts (CAFs) and myeloid-derived suppressor cells (MDSCs). CAFs contribute to immune evasion and tumor progression, and targeting them has shown promise in improving CAR-T efficacy (158). Similarly, the development of BCMA/CAF-CAR-T cells has demonstrated potential in reversing CAR-T dysfunction, a strategy that could benefit CAR-NK cells as well (159).

MDSCs are another key factor in the TME, known for their potent immunosuppressive effects on NK cell function, hindering CAR-NK cell cytotoxicity and promoting tumor growth. By inhibiting NK cell activity, MDSCs facilitate immune evasion, reducing the efficacy of CAR-NK cell therapies (160). Strategies to target MDSCs, such as depleting them or blocking their suppressive functions, could restore NK cell activity and improve therapeutic outcomes (161).

Moreover, targeting specific signaling pathways involved in MDSC-mediated suppression, such as reactive oxygen species (ROS) production or arginase activity, may enhance CAR-NK cell functionality (162). These approaches could help overcome MDSC-mediated immune suppression, creating a more favorable environment for CAR-NK cells.

The TME also harbors multiple immunosuppressive factors, such as hypoxia, transforming growth factor beta (TGF-β), prostaglandin E2 (PGE2), and extracellular metabolites like lactate and adenosine. These factors significantly restrain the activity of immune cells, including NK cells. For instance, TGF-β dampens various aspects of NK cell function, including cytokine secretion, degranulation, and mTOR signaling pathways, which are crucial for NK cell metabolism and functionality. Thus, targeting TGF-β represents a promising avenue for enhancing CAR-NK cell efficacy. Strategies such as knocking down TGF-β receptor II (TGF-βRII) on NK cells have shown promise in partially overcoming the negative influence of TGF-β without compromising anti-leukemia efficacy (163, 164).

Research is underway to explore various engineering approaches that could further bolster NK cell activity against the suppressive TME. For example, CAR structures that express negative TGF-β receptors or combine TGF-β receptor inhibitors with CAR-NK cell therapies may enhance their anti- tumor functions. Furthermore, tumor cells can upregulate ligands for inhibitory receptors on NK cells, leveraging the immune system’s negative feedback mechanisms to evade immune clearance. To counter this, strategies such as the knockdown of relevant inhibitory receptors like NKG2A on NK cells, or the use of specific checkpoint antibodies, can significantly improve anti-tumor effects (165, 166). Beyond TGF-β, extracellular adenosine poses another challenge within the TME. High concentrations of adenosine accumulate due to hypoxia-driven purinergic signaling, primarily generated by the activity of ectonucleotidases like CD39 and CD73. This accumulation exerts broad immunosuppressive effects on both NK and T cells, with CD39-expressing regulatory T cells (Tregs) capable of inhibiting NK cell anti-tumor activity. Therefore, employing ectonucleotidase inhibitors or A2 receptor antagonists could help mitigate the detrimental impact of adenosine on NK cells. Additionally, engineering NK cells to suppress the expression of adenosine receptors is another promising strategy (167–173). The concomitant use of checkpoint inhibitors is another strategy that may enhance the efficacy of CAR-NK therapies, as activated NK cells can express checkpoint molecules such as CTLA4 and PD-1 (174, 175). Disrupting these signaling pathways has been shown to enhance NK cell anti-cancer activity (176, 177). Moreover, other checkpoint inhibitors, including NKG2A, TIGIT, and TIM3, negatively impact NK function, and their inhibition could further improve the effectiveness of CAR-NK therapies (178–182).

### 6.4 Improving the targeted antitumor efficacy

The effectiveness of CAR-NK cell therapy depends heavily on the ability to identify and target appropriate antigens on tumor cells. Ideal CAR targets should exhibit high specificity, broad tumor cell type coverage, and consistent expression on cancer cells. However, achieving these attributes simultaneously remains a significant challenge. Currently, only a limited number of clinical targets meet these criteria, underscoring the need for innovative approaches to improve the antitumor capabilities of CAR-NK cells (183).

One promising approach to improving the effectiveness of CAR-NK cell therapy is the development of dual- or multi-specific CARs (184). These designs enable the targeting of multiple antigens simultaneously, which may help overcome tumor heterogeneity and reduce the likelihood of tumor escape. For instance, a clinical trial involving CD19/CD22-CAR-T cells for refractory acute lymphoblastic leukemia (ALL) has highlighted the potential of dual CAR strategies. In this trial, all patients achieved complete remission without minimal residual disease (MRD), underscoring the clinical efficacy of targeting multiple antigens. Such successes point to the possibility of translating similar strategies into CAR-NK cell therapy (185).

In another example, bispecific CAR-NK cells targeting both CD19 and B-cell maturation antigen (BCMA) exhibited strong cytotoxic activity against B-cell acute lymphoblastic leukemia (B-ALL) and multiple myeloma (MM) *in vitro*, suggesting the need for further *in vivo* studies to validate these findings (186). Additionally, triple-modified NK cells engineered with a CD19-specific CAR, the hnCD16 receptor, and an interleukin-15/interleukin-15 receptor fusion protein (IL-15RF) represent another innovative approach. This design aims to enhance antitumor potency and reduce the potential for tumor evasion (187). While boosting CAR-NK cell antitumor activity is vital, ensuring patient safety remains a critical consideration. CAR designs must strike a balance by effectively targeting tumor cells while minimizing the risk of “on-target, off-tumor” toxicity, where healthy tissues expressing the same target antigen as cancer cells may be inadvertently attacked. Research suggests that using CARs with reduced affinity for tumor-associated antigens (TAAs) can mitigate this risk. By ensuring activation of CAR-NK cells only when they encounter tumor cells with high antigen density, collateral damage to normal tissues can be minimized (188).

Another safety-enhancing strategy involves integrating suicide switches into CAR designs. A notable example is the inducible caspase 9 (iCasp9) system, which enables selective elimination of CAR-NK cells in cases of severe adverse events. This system has shown a favorable safety profile in preclinical and clinical studies, demonstrating its utility in managing potential toxicities while maintaining robust antitumor effects (20, 189, 190). Enhancing the targeted antitumor efficacy of CAR-NK cell therapy relies on the development of multi-specific CAR designs and the integration of safety mechanisms to reduce off-target effects. These advancements offer significant potential to improve therapeutic outcomes while ensuring a favorable safety profile, enabling broader clinical application of CAR-NK therapies.

### 6.5 Side effects of CAR-NK

Similar to other treatment methods, CAR-modified NK therapy is associated with some side effects. While CAR-T therapy has been known to produce a range of adverse effects, the most common and serious one is cytokine release syndrome (CRS). Other notable side effects of CAR-T therapy include “on-target, off-tumor” toxicity, neurotoxicity, tumor lysis syndrome (TLS), and graft-versus-host disease (GvHD) (191–194).

The risk of side effects in CAR-NK therapy is generally reduced due to two primary factors: the shorter lifespan of NK cells and their lower production of cytokines. Unlike CAR-T cells, CAR- modified NK cells have a limited lifespan and do not typically invade normal tissues such as the lungs and liver. Consequently, CAR-NK therapy tends to exhibit a more favorable safety profile, with lower incidences of GvHD and CRS. However, it is important to note that there has been at least one study reporting the occurrence of GvHD associated with CAR-NK cells (**Table 1**) (195).

**Table 1:** Advantages and limitations in CAR-T, CAR-NK, and CAR-M therapies.

Feature	CAR-T Cells	CAR-NK Cells	CAR-M Cells
Advantages	- Sufficient number of circulating T cells - Better persistence - Easier modification - Autologous setting (the patient is their own donor) - Previous studies on hematological malignancies facilitating its use on solid tumors	- Natural ability against non -self-cells - Lower cost - Direct and indirect killing functions due respectively to CAR and ADCC - Self-identification of normal cells by KIR - Better safety: Reduced risk of CRS, ICANS, GVHD, and neurotoxicity - Can be generated from multiple allogeneic sources (PBMC, UCB, NK-92 cell line, iPSC)	- Good infiltration into solid tumors - M1 macrophages feature a pro - Inflammatory phenotype - Antitumor activity by phagocytosis, presenting tumor antigen to Th1 cells, and production of anti - Inflammatory factors - Most abundant population in the TME of many cancer types - Important source of matrix metalloproteinase (MMP), which degrades almost all ECM - Can be generated from different sources
Limitations	- Tumor antigen heterogeneity and tumor antigen loss - Difficulty in infiltrating tumors - Limited survival and persistence in the immunosuppressive tumor microenvironment - CRS, OTOT toxicity, neurotoxicity, and GvHD	- Limited tumor infiltration - Limited efficacy in CAR transduction - Limited survival and persistence in the immunosuppressive tumor microenvironment	- Poor in vitro and in vivo proliferation - Limited efficacy in CAR transduction - Challenging genetic modification - CRS toxicity -OTOT toxicity - Need differentiation to M1 phenotype

• **ADCC (Antibody-Dependent Cellular Cytotoxicity):** A mechanism by which immune cells kill target cells that have been opsonized (tagged) with antibodies. • **KIR (Killer-Cell Immunoglobulin-like Receptor):** A receptor found on NK cells that helps them distinguish between normal and abnormal cells. • **CRS (Cytokine Release Syndrome):** A severe inflammatory response caused by excessive cytokine production following immune cell activation. • **ICANS (Immune Effector Cell-Associated Neurotoxicity Syndrome):** A neurological toxicity linked to CAR-T therapy. • **GVHD (Graft-versus-Host Disease):** A condition in which transplanted immune cells attack the recipient’s tissues. • **PBMC (Peripheral Blood Mononuclear Cells):** A type of blood cell used in immune therapies. • **UCB (Umbilical Cord Blood):** A source of hematopoietic stem cells for immune therapies. • **NK-92 Cell Line:** A standardized, highly cytotoxic NK cell line used for research and clinical applications. • **iPSC (Induced Pluripotent Stem Cells):** Stem cells generated from adult cells, which can differentiate into different cell types. • **M1 Macrophages:** A subset of macrophages with a pro-inflammatory and tumor-fighting phenotype. • **Th1 Cells (T-helper Type 1 Cells):** A subtype of T-helper cells that support cellular immune responses against pathogens and tumors. • **TME (Tumor Microenvironment):** The environment surrounding a tumor, including immune cells, blood vessels, and extracellular matrix. • **MMP (Matrix Metalloproteinases):** Enzymes that degrade extracellular matrix components, aiding in cell migration. • **ECM (Extracellular Matrix):** A network of proteins and molecules that provide structural support to cells. • **Immunosuppressive Tumor Microenvironment:** A tumor environment that inhibits immune cell function, allowing cancer cells to evade the immune system. • **CAR Transduction:** The process of genetically modifying immune cells to express a chimeric antigen receptor (CAR). • **In vitro & In vivo: In vitro** refers to studies conducted in a controlled environment outside a living organism (e.g., petri dish), while **in vivo** refers to studies conducted within a living organism. • **OTOT (On-Target, Off-Tumor) Toxicity:** A side effect where CAR-T or CAR-M cells attack normal cells that express the target antigen, leading to unintended damage. • **M1 Phenotype:** A specific functional state of macrophages that promotes inflammation and tumor destruction.

The most frequently observed side effects of CAR-NK therapy include fever and fatigue, which can arise from elevated serum levels of C-reactive protein (CRP) and interleukin-6 (IL-6). For example, the application of CD33-CAR-NK92 cells in patients with relapsed or refractory acute myeloid leukemia (AML) resulted in fever (reaching up to 40°C) and grade I CRS, accompanied by increased serum levels of IL-10 and IL-17. In contrast, CAR-NK therapy has not been associated with neurotoxicity or TLS in other studies. However, transient and reversible hematologic toxic effects have been documented (20, 109). The favorable safety profile of CAR-NK therapy encourages its continued application in clinical settings. Compared to CAR-T and CAR Macrophage (CAR-M) therapies, CAR-NK cells also exhibit reduced off-tumor toxicity and neurotoxicity risks, as highlighted in **Table 1**. Additionally, the limited lifespan of CAR-NK cells offers the opportunity for multiple infusions, which may lead to improved treatment outcomes. To further enhance the safety of CAR- modified NK therapy, methods such as the use of inducible suicide genes can be implemented, as discussed in further sections.

## 7 Future directions

The future of CAR-NK therapy is poised for significant advancements, building upon promising early results while addressing key challenges. A critical area of ongoing research is enhancing the persistence and expansion of NK cells *in vivo*, which is essential for improving the therapeutic efficacy of CAR- NK cells. Strategies being explored include incorporating cytokine support to sustain NK cell activity, utilizing gene editing techniques to knock out inhibitory receptors, and developing more sophisticated CAR designs that can boost NK cell potency (196).

The development of off-the-shelf CAR-NK products from allogeneic sources such as umbilical cord blood (UCB) and induced pluripotent stem cells (iPSCs) presents a groundbreaking opportunity for cancer immunotherapy. These allogeneic products could provide a readily available and cost-effective solution for cancer patients, circumventing the lengthy and expensive process of manufacturing patient-specific CAR-T cells. However, the viability and potency of these off-the-shelf products after freeze-thaw cycles, as well as their long-term durability, remain critical areas for further investigation (44). As CAR-NK therapy advances through clinical development, larger-scale trials will be essential to validate the safety, efficacy, and durability of the responses observed in early-phase studies. Such trials will help in understanding the long-term impacts of CAR-NK therapy, including potential side

effects and the optimal administration protocols. The integration of advanced genetic engineering and cell manufacturing technologies is anticipated to enhance the scalability and standardization of CAR- NK therapies (197). Moreover, the combination of CAR-NK therapies with other treatment modalities, such as checkpoint inhibitors or radiotherapy, may improve therapeutic outcomes by modulating the tumor microenvironment and enhancing immune responses. The exploration of bispecific CARs and tandem CARs can also facilitate the targeting of multiple tumor antigens simultaneously, further increasing the likelihood of effective tumor eradication (198). In conclusion, the evolution of CAR-NK therapy holds great promise as a safer, more versatile alternative to CAR- T cell therapy in the treatment of cancer. With ongoing advancements in research and technology, CAR-NK therapy has the potential to revolutionize cancer treatment, offering hope for improved patient outcomes and paving the way for broader applications in oncology

## 8 Conclusions

CAR-NK cell therapy represents a promising advancement in cancer immunotherapy, combining a superior safety profile with innate anti-tumor capabilities and the potential for scalable, allogeneic “off-the-shelf” applications. While initial clinical trials have demonstrated remarkable efficacy and safety in hematologic malignancies, the application of CAR-NK therapy in solid tumors is gaining momentum. Early trials targeting antigens such as HER2 in glioblastoma and ovarian cancer, as well as ROBO-1 in pancreatic cancer, have shown encouraging results, including tumor regression and minimal toxicities. These studies underscore CAR-NK’s potential to overcome the challenges posed by the immunosuppressive tumor microenvironment and heterogeneity in solid tumors.

However, critical challenges remain, including limited persistence, insufficient infiltration, and the need for optimized CAR designs specific to solid tumors. Innovative strategies such as engineering cytokine support, memory-like NK cells, and multi-specific CARs are actively being explored to address these barriers. As more clinical trials progress and expand into diverse tumor types, validation of CAR-NK therapy’s safety and efficacy will be pivotal.

With continued advancements in genetic engineering and integration with combination therapies, CAR-NK therapy is poised to revolutionize cancer treatment. Its adaptability and potential for broad application offer a transformative option for patients with aggressive and treatment-resistant solid tumors, bringing new hope to the field of oncology.
