# Supplementary material for: CAR-NK cell therapy: promise and challenges in solid tumors
Source: Front Immunol. 2025 Apr 7;16:1574742. doi: 10.3389/fimmu.2025.1574742 (PMC12009813; doi:10.3389/fimmu.2025.1574742)
Supplement: Supplementary file 1 [file Table1.docx]

| Status | Gov Identifier | Study Title | Conditions | Interventions | Phase/Ages/Gender | Study Design | Outcome Measures |
| --- | --- | --- | --- | --- | --- | --- | --- |
| Recruiting | NCT05410717 | Phase I/IIa Trial to Evaluate Safety and Preliminary Efficacy of CLDN6-CAR-NK in Patients With CLDN6-positive Advanced Solid Tumors | Stage IV Ovarian Cancer; Testis Cancer, Refractory; Endometrial Cancer, Recurrent | CAR-NK targeting Claudin6 CAR-NK cells | Phase 1, Phase 2 / 18-75 Years / All | Single Group Assignment / Open | Safety, Efficacy |
| Recruiting | NCT05213195 | NKG2D CAR-NK Cell Therapy in Patients With Refractory Metastatic Colorectal Cancer | Refractory Metastatic Colorectal Cancer | NKG2D CAR-NK | Phase 1 / 18-70 Years / All | Sequential Assignment / Open | Safety, ORR, DOR |
| Recruiting | NCT05528341 | NKG2D-CAR-NK92 Cells Immunotherapy for Solid Tumors | Relapsed/Refractory Solid Tumor | NKG2D-CAR-NK92 cells | Phase 1 / 18-75 Years / All | Single Group Assignment / Open | Safety, ORR, DCR |
| Recruiting | NCT05776355 | NKG2D CAR-NK & Ovarian Cancer | Ovarian Cancer | NKG2D CAR-NK | Not Applicable / 18+ Years / Female | Single Group Assignment / Open | Safety, DCR, ORR |
| Unknown | NCT05194709 | Study of Anti-5T4 CAR-NK Cell Therapy in Advanced Solid Tumors | Advanced Solid Tumors | Anti-CAR-NK Cells | Early Phase 1 / 18-80 Years / All | Single Group Assignment / Open | Safety, ORR, PFS |
| Unknown | NCT03692637 | Study of Anti-Mesothelin CAR-NK Cells in Epithelial Ovarian Cancer | Epithelial Ovarian Cancer | Anti-Mesothelin CAR-NK Cells | Early Phase 1 / 18-70 Years / Female | Single Group Assignment / Open | Occurrence of Adverse Events |
| Unknown | NCT05507593 | Study of DLL3-CAR-NK Cells in the Treatment of Extensive Stage Small Cell Lung Cancer | Extensive Stage SCLC | DLL3-CAR-NK cells | Phase 1 / 18-75 Years / All | Sequential Assignment / Open | DLT, MTD |
| Unknown | NCT03415100 | Pilot Study of NKG2D-Ligand Targeted CAR-NK Cells in Patients With Metastatic Solid Tumors | Solid Tumor | Biological: CAR-NK cells targeting NKG2D ligands | Phase 1 / 18-70 Years / All | Single Group Assignment / Open | Safety, Anti-tumor response |
| Unknown | NCT03940820 | Clinical Research of ROBO1 Specific CAR-NK Cells on Patients With Solid Tumors | Solid Tumor | ROBO1 CAR-NK cells | Phase 1, Phase 2 / 18-75 Years / All | Single Group Assignment / Open | Adverse events |
| Recruiting | NCT04847466 | Immunotherapy Combination: Irradiated PD-L1 CAR-NK Cells Plus Pembrolizumab Plus N-803 | Advanced Head and Neck Cancer | Drug: PD-L1 t-haNK | Phase 2 / 18+ Years / All | Single Group Assignment / Open | CR+PR, PFS |
| Recruiting | NCT05845502 | Single-arm, Open-label Clinical Study of SZ003 in the Treatment of Advanced Hepatocellular Carcinoma | Advanced Hepatocellular Carcinoma | SZ003 CAR-NK | Not Applicable / 18-80 Years / All | Single Group Assignment / Open | Adverse Events, ORR, OS |
| Unknown | NCT03941457 | Clinical Research of ROBO1 Specific BiCAR-NK Cells on Patients With Pancreatic Cancer | Pancreatic Cancer | BiCAR-NK cells (ROBO1 CAR-NK cells) | Phase 1, Phase 2 / 18-75 Years / All | Single Group Assignment / Open | Adverse events |
| Unknown | NCT03931720 | Clinical Research of ROBO1 Specific BiCAR-NK/T Cells on Patients With Malignant Tumor | Malignant Tumor | BiCAR-NK/T cells | Phase 1, Phase 2 / 18-75 Years / All | Single Group Assignment / Open | Adverse events |
| Unknown | NCT05686720 | Single-arm, Open-label Clinical Study of SZ011 in the Treatment of Advanced Triple Negative Breast Cancer | Advanced Triple Negative Breast Cancer | SZ011 CAR-NK | Early Phase 1 / 18-80 Years / Female | Single Group Assignment / Open | Adverse Events, ORR, PFS, OS |
| Unknown | NCT03692663 | Study of Anti-PSMA CAR-NK Cell (TABPEIC) in Metastatic Castration-Resistant Prostate Cancer | Metastatic Castration-Resistant Prostate Cancer | TABPEIC; Cyclophosphamide, Fludarabine | Early Phase 1 / 18+ Years / All | Single Group Assignment / Open | Treatment-related adverse events, PSA levels |
| Recruiting | NCT05856643 | Single-arm, Open-label Clinical Study of SZ011 in the Treatment of Ovarian Epithelial Carcinoma | Ovarian Epithelial Carcinoma | SZ011 CAR-NK | Early Phase 1 / 18-80 Years / Female | Single Group Assignment / Open | Adverse Events, ORR, PFS |
| Unknown | NCT05248048 | NKG2D CAR-T Cells to Treat Patients With Previously Treated Liver Metastatic Colorectal Cancer | Refractory Metastatic Colorectal Cancer | Biological: CAR-T infusion | Early Phase 1 / 18-75 Years / All | Single Group Assignment / Open | ORR, OS |
| Active, Not Recruiting | NCT03383978 | Intracranial Injection of NK-92/5.28.z Cells Combined With Intravenous Ezabenlimab in HER2-positive Glioblastoma | Glioblastoma | NK-92/5.28.z; Ezabenlimab | Phase 1 / 18+ Years / All | Single Group Assignment / Open | Adverse Events, Immune Response, ORR |
| Recruiting | NCT05703854 | Study of CAR.70-engineered IL15-transduced Cord Blood-derived NK Cells for Mesothelioma, Osteosarcoma | Mesothelioma, Osteosarcoma | CAR.70/IL15-transduced NK cells | Phase 1, Phase 2 / 18-80 Years / All | Single Group Assignment / Open | Safety, Adverse Events |
| Unknown | NCT02839954 | CAR-pNK Cell Immunotherapy in MUC1 Positive Relapsed or Refractory Solid Tumor | Hepatocellular Carcinoma, Lung Cancer, Pancreatic Carcinoma | Anti-MUC1 CAR-pNK cells | Phase 1, Phase 2 / 18+ Years / All | Single Group Assignment / Open | Adverse Events |
| Unknown | NCT05137275 | Study of Anti-5T4 CAR-raNK Cell Therapy in Locally Advanced or Metastatic Solid Tumors | Locally Advanced or Metastatic Solid Tumors | Anti-5T4 CAR-raNK Cells | Early Phase 1 / 18-80 Years / All | Single Group Assignment / Open | DLT, AEs, ORR, PFS |
| Recruiting | NCT03882840 | Induced-T Cell Like NK Cellular Immunotherapy for Cancer | Lack of MHC-I | ITNK cells | Phase 1, Phase 2 / 18+ Years / All | Single Group Assignment / Open | Safety, Best Response |
| Enrolling by Invitation | NCT03656705 | CCCR-NK92 Cells Immunotherapy for Non-small Cell Lung Cancer | Non-small Cell Lung Cancer | CCCR-NK92 cells | Phase 1 / 18-75 Years / All | Single Group Assignment / Open | Adverse Events, DCR, OS |
| Ongoing | ChiCTR2100048100 | Safety Study of Autologous MESO CAR NK Cells in Refractory Epithelial Ovarian Carcinoma | Epithelial Ovarian Carcinoma | MESO CAR NK | Phase 1 / 18-70 Years / Female | Single Arm / Open | Safety |
